# Role of p38_alpha/beta_ MAP Kinase in Cell Susceptibility to *Clostridium sordellii* Lethal Toxin and *Clostridium difficile* Toxin B

**DOI:** 10.3390/toxins9010002

**Published:** 2016-12-22

**Authors:** Ilona Schelle, Janina Bruening, Mareike Buetepage, Harald Genth

**Affiliations:** Institute for Toxicology, Hannover Medical School, Carl-Neuberg-Str. 1, D-30625 Hannover, Germany; ilona.schelle@t-online.de (I.S.); janina.bruening@twincore.de (J.B.); mbuetepage@ukaachen.de (M.B.)

**Keywords:** endocytosis, Ras, *C. difficile* Toxin B, mono-O-glucosylation, p21-activated kinase, actin

## Abstract

Lethal Toxin from *Clostridium sordellii* (TcsL), which is casually involved in the toxic shock syndrome and in gas gangrene, enters its target cells by receptor-mediated endocytosis. Inside the cell, TcsL mono-O-glucosylates and thereby inactivates Rac/Cdc42 and Ras subtype GTPases, resulting in actin reorganization and an activation of p38 MAP kinase. While a role of p38 MAP kinase in TcsL-induced cell death is well established, data on a role of p38 MAP kinase in TcsL-induced actin reorganization are not available. In this study, TcsL-induced Rac/Cdc42 glucosylation and actin reorganization are differentially analyzed in p38_alpha_^−/−^ MSCV empty vector MEFs and the corresponding cell line with reconstituted p38_alpha_ expression (p38_alpha_^−/−^ MSCV p38_alpha_ MEFs). Genetic deletion of p38_alpha_ results in reduced susceptibility of cells to TcsL-induced Rac/Cdc42 glucosylation and actin reorganization. Furthermore, SB203580, a pyridinyl imidazole inhibitor of p38_alpha/beta_ MAP kinase, also protects cells from TcsL-induced effects in both p38^−/−^ MSCV empty vector MEFs and in p38_alpha_^−/−^ MSCV p38_alpha_ MEFs, suggesting that inhibition of p38_beta_ contributes to the protective effect of SB203580. In contrast, the effects of the related *C. difficile* Toxin B are responsive neither to SB203580 treatment nor to p38_alpha_ deletion. In conclusion, the protective effects of SB203580 and of p38_alpha_ deletion are likely not based on inhibition of the toxins’ glucosyltransferase activity rather than on inhibited endocytic uptake of specifically TcsL into target cells.

## 1. Introduction

Toxin-producing strains of *C. difficile* and *C. sordellii* cause intestinal infections, including *C. difficile*-associated diarrhea (CDAD) in humans and horses, and *C. sordellii*-induced hemorrhagic enteritis and enterotoxemia in cattle, sheep, and other ruminants [1,2,3,4]. The major virulence factors involved in these infections are toxin A (TcdA) and toxin B (TcdB) of *C. difficile*, and lethal toxin (TcsL) and hemorrhagic toxin (TcsH) from *C. sordellii*. These single chained toxins exhibit an AB-like toxin structure with the C-terminal delivery domain mediating cell entry of the N-terminal glucosyltransferase domain by receptor-mediated endocytosis [5,6]. The endocytosed glucosyltransferase domain associates with membrane phosphatidylserine facilitating mono-O-glucosylation of small GTPases of the Rho and Ras subfamilies in a monovalent and divalent metal ion-dependent manner [7,8,9]. The acceptor amino acid of toxin-catalyzed mono-O-glucosylation is Thr-35 in Rac1, Cdc42, and (H/K/N)Ras and Thr-37 in Rho(A/B/C). Mono-O-glucosylated Rho/Ras GTPases are incapable of coupling to their regulatory and effector protein and thus are functionally inactive [10,11,12,13]. Treatment of cultured cells with the glucosylating toxins results in a breakdown of the actin-based cytoskeleton (“cytopathic effect”) and (at higher toxin concentrations) in cell death (“cytotoxic effect”) based on inhibition of Rho/Ras-dependent signaling pathways regulating actin dynamics [14], cell-matrix binding [15,16], cell cycle progression, and cell survival [17,18,19].

The family of p38 MAPKs encompasses the four isoforms p38_alpha_, p38_beta_, p38_gamma_, and p38_delta_. p38_alpha_ and p38_beta_ are ubiquitously expressed, while p38_gamma_ and p38_delta_ exhibit a more restricted expression patterns. The best characterized isoform is p38_alpha_, which is involved in the regulation of the main cellular functions, including actin dynamics, differentiation, and cell death and survival [20,21]. TcsL (as well as TcdA and TcdB) has been shown to activate the family of mitogen-activated protein kinases (MAPKs) involving ERKs (extracellular signal-regulated kinases), JNKs (c-Jun-*N*-terminal kinases) and p38 MAPKs [22]. MAP kinase signaling cascades have been shown to be involved in the cytopathic as well as the cytotoxic effects of the glucosylating toxins. In particular, TcsL-induced activation of JNK has been suggested to facilitate TcsL-catalyzed GTPase substrate glucosylation and subsequently TcsL-induced actin reorganization [22]. p38_alpha_ MAP kinase signaling has been implicated in TcsL-induced expression of the cell death-regulating GTPase RhoB [23]. Finally, TcsL-induced cell death has been shown to be responsive to inhibition by SB203580, a pyridinyl imidazole inhibitor of p38_alpha/beta_ MAP kinase, suggesting a role of p38_alpha/beta_ in TcsL-induced cell death [24].

In this study, TcsL-induced Rac/Cdc42 glucosylation and subsequent actin reorganization were analyzed for an involvement of p38_alpha_ in murine embryonic fibroblasts (MEFs). In this study, TcsL-induced Rac/Cdc42 glucosylation and actin reorganization are differentially analyzed in p38_alpha_^−/−^ murine stem cell virus empty vector MEFs (p38_alpha_^−/−^ MSCV EV MEFs) and the corresponding cell line with reconstituted p38_alpha_ expression (p38_alpha_^−/−^ MSCV p38_alpha_ MEFs) [25,26]. We show that genetic deletion of p38_alpha_ as well as SB203580 protects cells from TcsL-catalyzed glucosylation of Rac/Cdc42 subtype GTPases and TcsL-induced actin reorganization.

## 2. Results

### 2.1. Prevention of TcsL-Induced Actin Re-Organization upon Inhibition of p38_alpha/beta_

**Figure 1:**
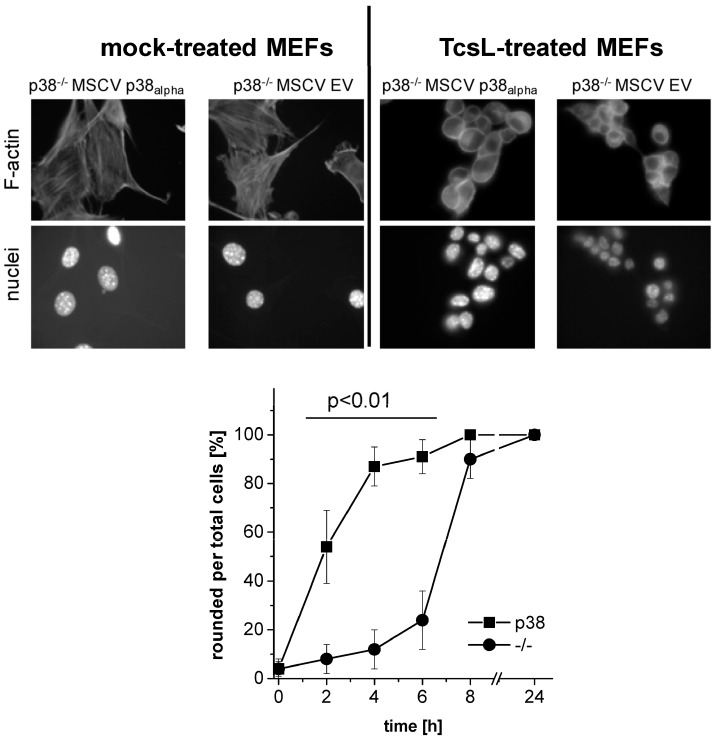
Effects of genetic deletion of p38_alpha_ on TcsL-induced changes of cell morphology (time-dependency). p38_alpha_^−/−^ MSCV empty vector (EV) MEFs and the corresponding cell line with reconstituted p38_alpha_ expression (p38_alpha_^−/−^ MSCV p38_alpha_ MEFs) were treated with TcsL (1 µg/mL) for the indicated times. Cells were then washed, fixed, permeabilized, and stained with rhodamine-phalloidin and DAPI. Cell morphology was visualized using fluorescence microscopy (20× amplification). TcsL-induced changes of the morphology were time-dependently quantified in terms of the number of rounded per total cells. Six representative microscopic fields were chosen and 300 cells total were counted for characteristic cell rounding. Values are the mean ± SD from three independent experiments performed in triplicates. *p* < 0.01 indicates significant differences comparing p38_alpha_-proficient with p38_alpha_-deficient cells using Student’s *t*-test.

**Figure 2:**
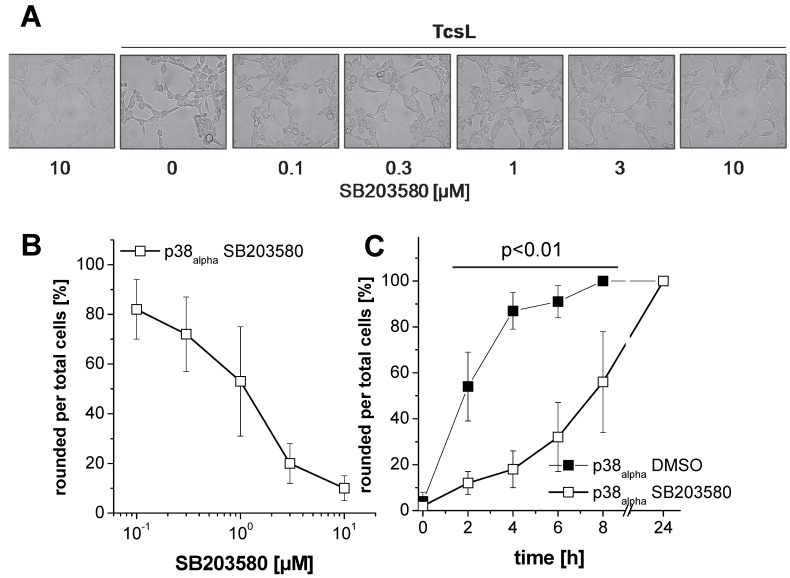
Effects of SB203580 treatment on TcsL-induced changes of cell morphology: (**A**) p38_alpha_^−/−^ MSCV p38_alpha_ MEFs were treated with TcsL (1 µg/mL) or buffer in the presence of the indicated concentrations of SB203580 for 4 h. Cell morphology was visualized using phase contrast microscopy (10× amplification). (**B**) TcsL-induced changes of the morphology were quantified in terms of the number of rounded per total cells. Six representative microscopic fields were chosen and 300 cells total were counted for characteristic cell rounding. Values are the mean ± SD from three independent experiments performed in triplicates. (**C**) p38_alpha_^−/−^ MSCV p38_alpha_ MEFs were treated with TcsL (1 µg/mL) in the presence of SB203580 (10 µM) or DMSO alone for the indicated times. TcsL-induced changes of the morphology were quantified in terms of the number of rounded per total cells. Values are the mean ± SD from three independent experiments performed in triplicates. *p* < 0.01 indicates significant differences comparing SB203580-treated with DMSO-treated cells using Student’s *t*-test.

**Figure 3:**
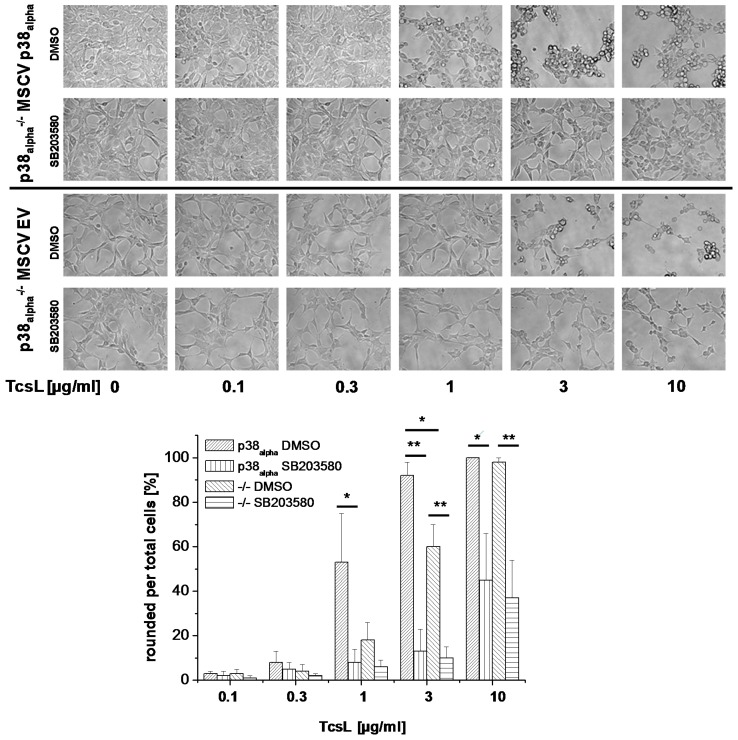
Effects of genetic deletion of p38_alpha_ and of SB203580 treatment on TcsL-induced changes of cell morphology (concentration-dependency). p38_alpha_^−/−^ MSCV p38_alpha_ MEFs and p38_alpha_^−/−^ MSCV empty vector (EV) MEFs were treated with the indicated concentrations of TcsL in the presence of SB203580 (10 µM) or DMSO alone for 4 h. Cell morphology was visualized using phase contrast microscopy (10× amplification). TcsL-induced changes of the morphology of p38^−/−^ MSCV p38_alpha_ MEFs and of p38^−/−^ MSCV empty vector MEFs were quantified in terms of the number of rounded per total cells. Six representative microscopic fields were chosen and 300 cells total were counted for characteristic cell rounding. Values are the mean ± SD from three independent experiments performed in triplicates. */** indicates significant differences, *p* < 0.05/*p* < 0.005 as analyzed using Student’s *t*-test.

TcsL time-dependently induced actin reorganization in p38_alpha_-proficient p38_alpha_^−/−^ MSCV p38_alpha_ MEFs (Figure 1), with TcsL treatment for 4 h being sufficient for almost complete cell rounding (Figure 1). In contrast, TcsL-induced rounding of p38_alpha_-deficient p38_alpha_^−/−^ MSCV EV MEFs was clearly delayed, suggesting a reduced susceptibility of p38_alpha_^−/−^ MSCV EV MEFs to TcsL. Next, TcsL-induced cell rounding was analyzed in p38_alpha_^−/−^ MSCV p38_alpha_ MEFs treated with SB203580, a pyridinyl imidazole inhibitor of p38_alpha/beta_ MAP kinase [27]. SB203080 concentration-dependently reduced TcsL-induced cell rounding, with a SB203580 concentration of 10 µM being sufficient for almost complete prevention of TcsL-induced cell rounding (Figure 2A,B). A pronounced protective effect of SB203580 was also observed in a time-dependent experiment (Figure 2C). SB203580 (10 µM) alone did not change fibroblast morphology (Figure 2A). Finally, the protective effect of p38_alpha_ inhibition was analyzed in TcsL concentration-dependent experiments (Figure 3). Either genetic deletion of p38_alpha_ or SB203580 treatment delayed TcsL-induced cell rounding. Interestingly, SB203580 treatment of p38_alpha_-deficient p38_alpha_^−/−^ MSCV EV MEFs further delayed TcsL-induced cell rounding, suggesting a role of p38_beta_ inhibition in the reduced susceptibility to TcsL.

**Figure 4:**
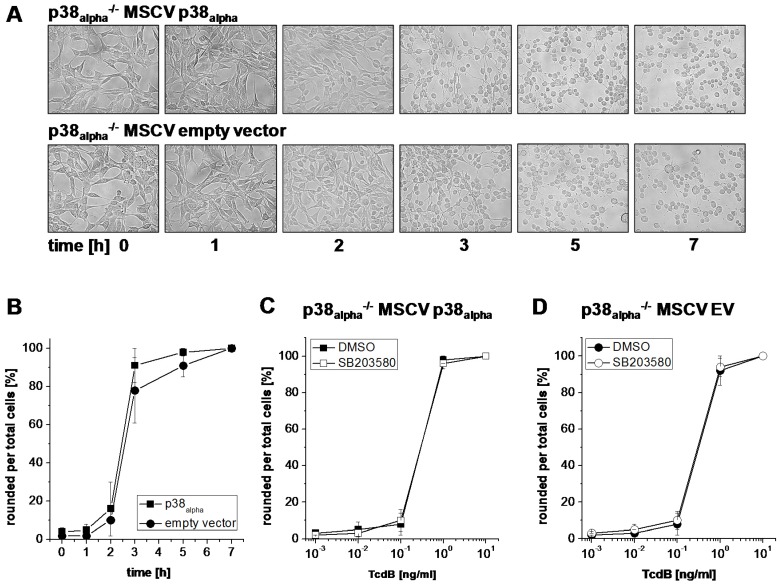
Effects of genetic deletion of p38_alpha_ and of SB203580 treatment on TcdB-induced changes of cell morphology: (**A**,**B**) p38_alpha_^−/−^ MSCV p38_alpha_ MEFs and p38_alpha_^−/−^ MSCV empty vector (EV) MEFs were treated with TcdB (1 ng/mL) for the indicated times; and (**C**,**D**) p38_alpha_^−/−^ MSCV p38_alpha_ MEFs and p38_alpha_^−/−^ MSCV empty vector MEFs were treated with the indicated concentrations of TcdB in the presence of SB203580 (10 µM) or DMSO alone for 4 h. Cell morphology was visualized using phase contrast microscopy (10× amplification). TcdB-induced changes of the morphology were quantified in terms of the number of rounded per total cells. Six representative microscopic fields were chosen and 300 cells total were counted for characteristic cell rounding. Values are the mean ± SD from three independent experiments performed in triplicates.

TcdB is highly related to TcsL (identity of 75% at amino acid level), as both TcdB and TcsL enter their target cells by receptor-mediated endocytosis and both cause changed actin dynamics by mono-O-glucosylation of small GTPases. Interestingly, neither genetic deletion (Figure 4A,B) nor treatment with the p38_alpha/beta_ inhibitor SB203580 (Figure 4C,D) changed the kinetics of TcdB-induced actin reorganization. p38_alpha/beta_ inhibition thus mediates protection of fibroblasts from TcsL-(not TcdB-) induced actin reorganization.

### 2.2. Prevention of TcsL-Induced Glucosylation of Rac/Cdc42 Subtype GTPases upon Inhibition of p38_alpha/beta_

**Figure 5:**
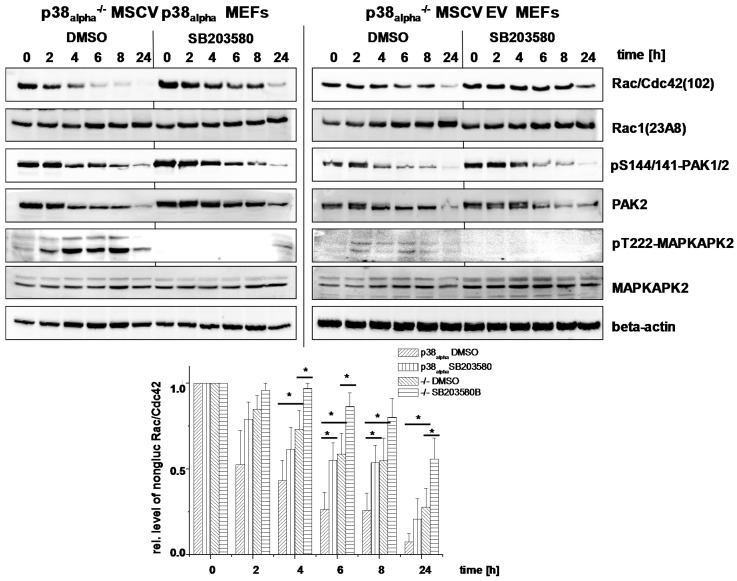
Effects of genetic deletion of p38_alpha_ and of SB203580 treatment on TcsL-catalyzed Rac/Cdc42 glucosylation (time-dependency). p38_alpha_^−/−^ MSCV p38_alpha_ MEFs and p38_alpha_^−/−^ MSCV empty vector (EV) MEFs were treated with TcsL (1 µg/mL) in the presence of SB203580 (10 µM) or DMSO alone for the indicated times. The cellular levels of non-glucosylated Rac/Cdc42, total Rac1, pS144/141-PAK1/2, PAK2, pT222-MAPKAPK2, MAPKAPK2, and beta-actin were analyzed by immunoblotting using the indicated antibodies. Quantifications of immunoblots were performed using Kodak software and relative amounts of non-glucosylated Rac/Cdc42 versus the total levels of Rac1, respectively, are expressed as mean ± SD of three independent experiments. * indicates significant differences, *p* < 0.05, as analyzed using Student’s *t*-test.

**Figure 6:**
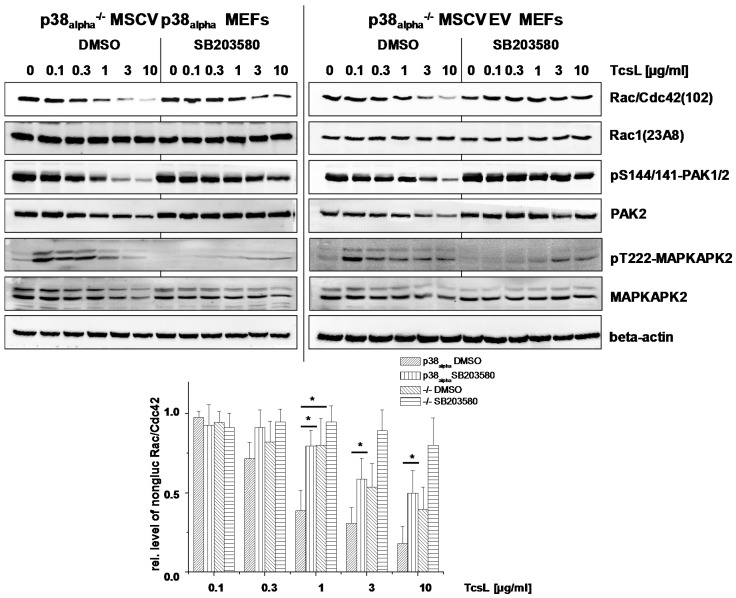
Effects of genetic deletion of p38_alpha_ and of SB203580 treatment on TcsL-catalyzed Rac/Cdc42 glucosylation (TcsL concentration-dependency). p38_alpha_^−/−^ MSCV p38_alpha_ MEFs and p38_alpha_^−/−^ MSCV empty vector (EV) MEFs were treated with the indicated concentrations of TcsL in the presence of SB203580 (10 µM) or DMSO alone for 4 h. The cellular levels of non-glucosylated Rac/Cdc42, total Rac1, pS144/141-PAK1/2, PAK2, and beta-actin were analyzed by immunoblotting using the indicated antibodies. Quantifications of immunoblots were performed using Kodak software and relative amounts of non-glucosylated Rac/Cdc42 versus the total levels of Rac1, respectively, are expressed as mean ± SD of three independent experiments. * indicates significant differences, *p* < 0.05, as analyzed using Student’s *t*-test.

**Figure 7:**
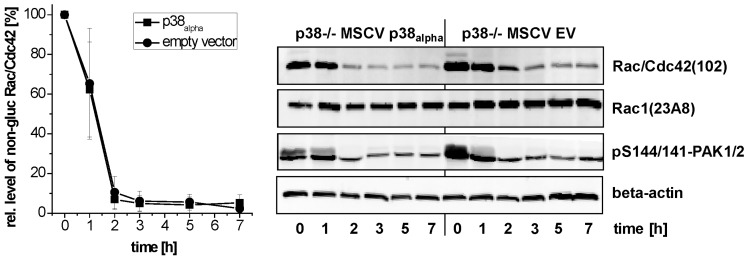
Effects of genetic deletion of p38_alpha_ on TcdB-catalyzed Rac/Cdc42 glucosylation (time-dependency). p38_alpha_^−/−^ MSCV p38_alpha_ MEFs and p38_alpha_^−/−^ MSCV empty vector (EV) MEFs were treated with TcdB (1 ng/mL) for the indicated times. The cellular levels of non-glucosylated Rac/Cdc42, total Rac1, pS144/141-PAK1/2, and beta-actin were analyzed by immunoblotting using the indicated antibodies. Quantifications of immunoblots were performed using Kodak software and relative amounts of non-glucosylated Rac/Cdc42 versus the total levels of Rac1, respectively, are expressed as mean ± SD of three experiments.

Actin reorganization has been attributed to TcsL-/TcdB-catalyzed glucosylation of Rac/Cdc42 subtype GTPases and the subsequent loss of cell-matrix binding [15,28,29]. For the analysis of TcsL-catalyzed glucosylation of Rac/Cdc42, lysates from p38_alpha_^−/−^ MSCV p38_alpha_ MEFs were analyzed by immunoblot analysis using the Rac1(clone 102) antibody. This antibody is specific for non-glucosylated Rac/Cdc42 subtype GTPases [30,31]. Once Rac/Cdc42 subtype GTPases are glucosylated by TcsL, the antibody does not detect its epitope, resulting in a lost signal. TcsL-treated p38_alpha_^−/−^ MSCV p38_alpha_ MEFs exhibited time-dependent glucosylation of Rac/Cdc42 subtype GTPases (Figure 5A). The cellular level of Rac1 was not changed upon TcsL treatment (as analyzed using the Rac1(clone 23A8) antibody) (Figure 5A), confirming that decreasing detection of Rac/Cdc42 subtype GTPases by the Rac1(Mab 102) antibody was due to glucosylation but not due to degradation. TcsL-catalyzed Rac/Cdc42 glucosylation results in dephosphorylation of the FA component p21-associated kinase1/2 (PAK1/2), which is a Rac/Cdc42 effector protein [15,16]. TcsL treatment time-dependently resulted in decreasing levels of pS144/141-PAK1/2 (Figure 5A), indicating PAK1/2 deactivation [32]. The levels of total PAK2 also time-dependently decreased in TcsL-treated cells (Figure 5), showing that PAK1/2 deactivation was based on both dephosphorylation and degradation. TcsL-catalyzed Rac/Cdc42 glucosylation (i.e., inactivation) was thus reflected by deactivation of its effector kinase PAK1/2. Finally, p38 MAP kinase activity was tracked in terms of phosphorylation of its downstream target MAPKAPK2. TcsL treatment resulted in a transient increase of pT222-MAPKAPK2, indicating transient MAPKAPK2 activation. In p38_alpha_-deficient p38^−/−^ MSCV empty vector MEFs, in which MAPKAPK2 activation was hardly observed, Rac/Cdc42 glucosylation was clearly delayed as compared with p38_alpha_^−/−^ MSCV p38_alpha_ MEFs (Figure 5). In a TcsL concentration-dependent experiment, TcsL turned out to be at least three fold more potent in inducing Rac/Cdc42 glucosylation (Figure 6) and PAK1/2 deactivation (Figure 6) in p38^−/−^ MSCV p38_alpha_ MEFs as compared with p38^−/−^ MSCV empty vector MEFs. In contrast to TcsL, the kinetics of TcdB-induced Rac/Cdc42 glucosylation and of PAK1/2 deactivation were comparable in p38_alpha_^−/−^ MSCV p38_alpha_ and p38^−/−^ MSCV empty vector MEFs (Figure 7). TcdB-catalyzed Rac/Cdc42 glucosylation was thus not susceptible to genetic p38_alpha_ inhibition (Figure 7), consistent with above observations on TcdB-induced actin reorganization (Figure 4). These observations show that genetic p38_alpha_ inhibition protects fibroblasts specifically from TcsL-(not TcdB-) induced cytopathic effects.

TcsL-induced Rac/Cdc42 glucosylation were next analyzed upon pharmacological inhibition of p38_alpha/beta_. SB203580 treatment of either p38_alpha_^−/−^ MSCV p38_alpha_ and p38_alpha_^−/−^ MSCV empty vector MEFs resulted in an almost complete loss of pT222-MAPKAPK2, confirming effective p38 inhibition (Figure 5 and Figure 6). TcsL-catalyzed Rac/Cdc42 glucosylation and PAK1/2 deactivation were responsive to SB203580 treatment in both p38_alpha_^−/−^ MSCV p38_alpha_ and p38_alpha_^−/−^ MSCV empty vector MEFs, both in time- (Figure 5) and concentration-dependent (Figure 6) experiments. The observation that cell rounding and Rac/Cdc42 glucosylation in p38_alpha_^−/−^ MSCV empty vector MEFs are responsive to SB203580 suggests that the protective effects of SB203580 involve inhibition of both p38_alpha_ and p38_beta_. Taken together, p38_alpha/beta_ inhibition-mediated protection of fibroblasts from TcsL-induced actin reorganization coincides with protection from TcsL-catalyzed Rac/Cdc42 glucosylation.

### 2.3. SB203580 Preserves TcsL-Induced Loss of Epithelial Barrier Function

**Figure 8:**
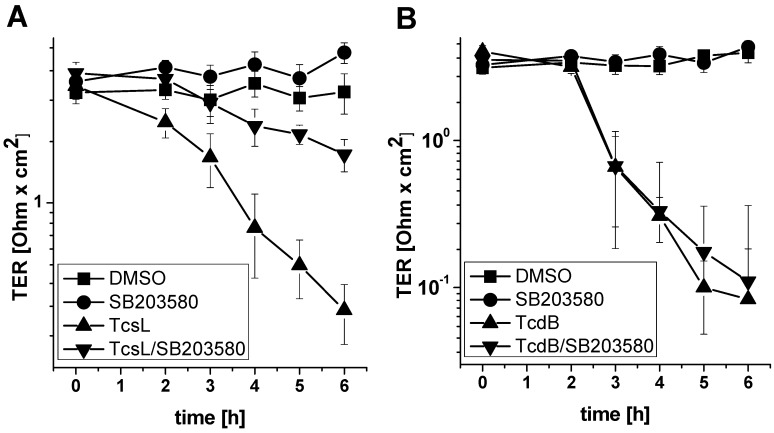
SB203580 preserves TcsL-induced loss of epithelial barrier function. Madin-Darby canine kidney (MDCK-C7) monolayers grown on Transwell filter inserts were treated with TcsL (30 µg/mL (**A**)) and TcdB (30 ng/mL (**B**)) in the presence of SB203580 (10 µM) or DMSO alone as indicated. Transepithelial electrical resistance (TER) was monitored for the indicated times. TER values are given as means ± SD of three independent experiments.

*C. sordellii*-associated disease include necrotic and hemorrhagic enteritis, whereby TcsL has been shown to alter epithelial permeability [33,34]. To check if SB203580-mediated inhibition of TcsL might be useful with regard to disease treatment, TcsL-induced loss of epithelial barrier function was next analyzed in terms of the loss of the transepithelial resistance (TER) of a Madin-Darby canine kidney (MDCK-C7) monolayer [9,35]. TcsL treatment time-dependently decreased the TER of the MDCK-C7 monolayer (Figure 8A). In the presence of SB203580, TcsL-induced loss of TER was markedly attenuated (Figure 8A). SB203580 alone did not affect the TER (Figure 8A). In contrast, TcdB-induced loss of the TER of the MDCK-C7 monolayer was not responsive to SB203580 treatment (Figure 8B), consistent with above observations showing that TcdB-induced actin reorganization was not responsive to SB203580 treatment (Figure 4). SB203580 treatment thus might be useful in the light of treatment of the TcsL-induced loss of epithelial barrier function.

## 3. Discussion

In this study, SB203580, a pyridinyl imidazole inhibitor of p38_alpha/beta_ MAP kinase, has been presented to efficaciously prevent TcsL-induced loss of epithelial barrier function of MDCK-C7 monolayers and to prevent TcsL-induced cell rounding, Rac/Cdc42 glucosylation, and PAK deactivation in murine fibroblasts. Furthermore, genetic deletion of p38_alpha_ is also sufficient for preventing TcsL-induced cell rounding, Rac/Cdc42 glucosylation, and PAK deactivation in murine fibroblasts. Interestingly, p38_alpha_^−/−^ MSCV empty vector MEFs turn out to be still sensitive to SB203580, suggesting that the protective effects of SB203580 involves inhibition of both p38_alpha_ and p38_beta_. How does p38 inhibition mediate the protective effect against TcsL?

TcdB and TcsL both are mono-O-glucosyltransferases that modify small GTPases. Their N-terminal glucosyltransferase domains are structurally and functionally highly related, as they share Rac/Cdc42 as substrate GTPases (with threonine-35 being the acceptor amino acid) and UDP-glucose as a sugar donor [36]. From the observation that TcdB-induced Rac/Cdc42 glucosylation is insensitive to p38 inhibition, it can be concluded that SB203580 does not interfere with the intracellular glucosyltransferase activity of the toxins. This leads to the new hypothesis that p38_alpha/beta_ inhibition affects endocytic uptake of TcsL into target cells. In fact, members of the p38 MAP kinase family are important regulators of endocytosis, as they control endocytic trafficking via the GDI-Rab5 complex [37]. In particular, p38 MAP kinase regulates stress-induced internalization of the epidermal growth factor receptor (EGFR) and μ opioid receptor endocytosis [38,39,40]. Against this background, inhibition of p38_alpha/beta_ might prevent cell entry of specifically TcsL (not TcdB) by receptor-mediated endocytosis. This hypothetic model implies that TcsL and TcdB enter their target cells by exploiting distinct cell surface receptors, with the TcsL cell surface receptors being internalized in a p38_alpha/beta_-dependent and the TcdB cell surface receptors being internalized in a p38_alpha/beta_-independent manner.

In a former study, the responsiveness of TcsL-induced effects such as apoptotic cell death to inhibition by SB203580 has been interpreted in terms of an involvement of p38_alpha/beta_ in TcsL-induced cell death [24]. The observations of this study have led to the conclusion that the protective effect of SB203580 is based on rather inhibition of TcsL uptake than on a role of p38 in the cytopathic effects of TcsL. Against this background, the responsiveness of TcsL-induced cell death to inhibition by SB203580 must be re-interpreted in terms of reduced TcsL uptake and subsequently reduced GTPase substrate glucosylation as the cause of cell death inhibition. In autophagy research, the pyridinyl imidazole inhibitors SB203580 and SB202190 have been shown to interfere with the autophagic flux independently of p38 MAP kinase [41]. These observations have led to the recommendation that pyridinyl imidazole class inhibitors should not be used as pharmacological tools in the analysis of MAPK11-MAPK14/p38-dependence [42]. The latter recommendation seems to be applicable also in the field of protein toxins.

The protective effect of the pyridinyl imidazole SB203580 is most interesting with regard to the development of non-antibiotic treatment for diseases caused by toxigenic *C. sordellii*. Further research will address the characterization of those pathways mediating the protective effect against TcsL upon inhibition of p38_alpha/beta_. Furthermore, a screening of additional pyridinyl imidazole compounds capable of inhibiting the effects of TcsL is under way in our laboratory.

## 4. Conclusions

- Genetic deletion of p38_alpha_ or treatment with SB203580 protects MEFs from Rac/Cdc42 glucosylation and actin reorganization induced by TcsL (not by the related TcdB).
- Treatment with SB203580 protects epithelial monolayer from loss of epithelial barrier function induced by TcsL (not by TcdB).
- The protective effects of SB203580 treatment and of p38_alpha_ deletion are likely based on inhibition of endocytic uptake of TcsL rather than on inhibition of the toxins’ glucosyltransferase activity.

## 5. Materials and Methods

### 5.1. Materials

The following reagents were obtained from commercial sources: SB203580 (4-(4-fluorophenyl)-2-(4-methylsulfinylphenyl)-5-(4-pyridyl)imidazole) (Calbiochem, Darmstadt, Germany); DAPI (40.6-diamidino-2-phenylindole) (Sigma-Aldrich, St. Louis, MO, USA); and rhodamine-conjugated phalloidin (Sigma-Aldrich, St. Louis, MO, USA).

Toxins: TcsL was prepared from *C. sordellii* IP82, which is the same strain as 6018, and TcdB from *C. difficile* VPI10463. Toxins were produced and purified yielding only one band on SDS-PAGE as previously described [43,44]. In brief, a dialysis bag containing 900 mL of 0.9% NaCl in a total volume of 4 liters of brain heart infusion (Difco, BD Life Sciences, Heidelberg, Germany) was inoculated with 100 mL of an overnight culture of *C. sordellii* or *C. difficile*. The culture was grown under microaerophilic conditions at 37 °C for 72 h. Bacteria were removed from the dialysis bag solution by centrifugation. Proteins from the culture supernatant from were precipitated by ammonium sulfate (Merck Millipore, Darmstadt, Germany) at 70% saturation. The precipitated proteins were dissolved in 50 mM Tris-HCl pH 7.5 buffer and extensively dialyzed against 50 mM Tris-HCl pH 7.5 buffer for 24 h. The protein solution was loaded onto an anion exchange column (MonoQ, GE Healthcare Europe, Freiburg, Germany). Either TcsL or TcdB were eluted with 50 mM Tris-HCl, pH 7.5, at 500–600 mM NaCl and were subsequently dialyzed against buffer (50 mM Tris-HCl pH 7.5, 15 mM NaCl). The absence of TcdA (which eluted at 150–200 mM NaCl) in TcdB preparations was checked by immunoblot analysis.

### 5.2. Cell Culture and Preparation of Lysates

p38^−/−^ MSCV empty vector MEFs and the corresponding p38^−/−^ MSCV p38_alpha_ MEFs (kindly provided by Dr. Angel Nebreda, Institute for Research in Biomedicine, Barcelona, Spain) were cultivated in Dulbecco’s modified essential medium supplemented with 10% FCS, 100 µg/mL penicillin, 100 U/mL streptomycin and 1 mM sodium pyruvate at 37 °C and 5% CO_2_ according to standard protocols [45]. Cells sub-confluently seeded in 3.5-cm dishes were treated with TcsL, TcdB, and SB203580 for different times and concentrations as noted in the figures. Thereby, cells were pretreated with 10 µM SB202580 dissolved in DMSO (final DMSO concentration in the medium 2%) for 20 min and subsequently treated with the toxins or buffer. Upon incubation time, the cells were rinsed with 5 mL of ice-cold phosphate-buffered saline and scraped off in 200 µL of Laemmli lysis buffer per dish. The cells were disrupted mechanically by sonification (five times on ice). The lysate were submitted to immunoblot analysis.

### 5.3. Immunoblot Analysis

Cells lysates were separated on 15% polyacrylamide gels and transferred onto nitrocellulose for 2 h at 250 mA, followed by blocking with 5% (*w*/*v*) nonfat dried milk for 1 h. Blots were incubated with the appropriate primary antibody with dilution according to the manufacturers’ instructions (beta-actin, Mab AC-40, Sigma-Aldrich, St. Louis, MO, USA; dilution 1:5000); MAPKAPK-2 (Cell signaling 3042, dilution 1:1000); pT222-MAPKAPK-2 (Cell signaling 3316, dilution 1:1000); PAK2 (Cell signaling 2608, dilution 1:1000); phospho-S144/141-PAK1/2 (Abcam ab40795; dilution 1:2000); Rac1 (BD Transduction Laboratories 610650, clone 102; dilution 1:1000); Rac1(Millipore 05-389, clone 23A8; dilution 1:1000) in buffer B (50 mM Tris-HCl, pH 7.2, 150 mM NaCl, 5 mM KCl, 0.05% (*w*/*v*) Tween 20) for 18 h and subsequently for 2 h with a horseradish peroxidase-conjugated secondary antibody (mouse: Rockland 610-1034-121; dilution 1:3000; rabbit Rockland 611-1302; dilution 1:3000). For the chemiluminescence reaction, ECL Femto (Fisher Scientific, Schwerte, Germany) was used. The signals were analyzed densitometrically using the KODAK 1D software (2004, Rochester, MN, USA).

### 5.4. Visualization of the Actin Cytoskeleton

p38_alpha_^−/−^ MSCV empty vector MEFs and the corresponding p38_alpha_^−/−^ MSCV p38_alpha_ MEFs were grown on coverslips, fixed with formaldehyde (4%) in PBS and permeabilized with 0.1% Triton X-100 (Sigma-Aldrich, St. Louis, MO, USA). The actin cytoskeleton and the nuclei were labeled using rhodamine-phalloidin and DAPI, respectively. Fluorescence images were recorded using a Zeiss Axiovert M (Jena, Germany).

### 5.5. Transepithelial Resistance of Epithelial Monolayers

Madin-Darby canine kidney (MDCK-C7) cells were cultured under standard conditions (37 °C, 5% CO_2_) as described [35]. Briefly, MDCK-C7 cells were cultured in minimum essential medium (MEM) enriched with Earle’s salts, non-essential amino acids, glutamic acid and 10% fetal calf serum (Biochrom, Berlin, Germany) and split twice weekly using standard culture techniques. MDCK-C7 cells were seeded onto 12 well filter transwell inserts (pore size 0.4 µM, BD Life Sciences, Heidelberg, Germany). The transepithelial electrical resistance (TER) was determined by a Voltohmmeter equipped with Endom 24 chamber (EVOM, World Precision Instruments, Berlin, Germany). MDCK-C7 monolayers were cultivated up to an initial resistance of >2 kΩ·cm^2^. The toxins and SB203580 (final DMSO concentration in the medium 2%) were applied on the basolateral site of the monolayer and toxin-induced loss of TER was analyzed in a time-dependent manner.
